# Biomechanical Effects of Stemmed Total Knee Arthroplasty on the Human Femur: A CT-Data Based Study

**DOI:** 10.1155/2022/5738610

**Published:** 2022-09-12

**Authors:** Elisabeth M. Sporer, Christoph Schilling, Adrian Sauer, Robert J. Tait, Alexander Giurea, Thomas M. Grupp

**Affiliations:** ^1^Aesculap AG, Research & Development, 78532 Tuttlingen, Germany; ^2^Ludwig Maximilians University Munich, Medical Department, 80336 Munich, Germany; ^3^Ludwig Maximilians University Munich, Department of Orthopaedic and Trauma Surgery, Musculoskeletal University Center Munich (MUM), 81377 Munich, Germany; ^4^Orthopaedic Institute of Henderson, Henderson, Nevada 89052, USA; ^5^Medical University of Vienna, Dept. of Orthopaedic Surgery, 1090 Vienna, Austria

## Abstract

End-of-stem pain of the femur is a common problem in revision total knee arthroplasty (TKA). It may be caused by a problematic interaction between stem and bone, but the exact biomechanical correlate is still unknown. The aim of this prospective study was to find out how the stem is positioned in the medullary canal, how the femoral geometry changes due to implantation, and whether the results are influenced by the diameter of the trial. We implanted 16 rotating hinge knee implants into 16 fresh-frozen human femora using the hybrid fixation technique and comparing two reaming protocols. We created 3-dimensional models of the specimens before and after implantation using CT-scans and calculated the differences. The main contact between stem and bone was found at the proximal 30 mm of the stem, especially anterior. We observed two different contact patterns of stem and bone. The cortical thickness was reduced especially at the anterior tip of the stem with a maximum reduction of 1405 ± 501 *μ*m in the standard group and 980 ± 447 *μ*m in the small_trial group, which is a relative reduction of 34 ± 14% (standard group) and 26 ± 14% (small_trial group). The bone experienced a deformation to posterior and lateral. We conclude that the tip of the stem is an important biomechanical region. Different contact patterns between stem and bone as well as the reduction in cortical thickness at the tip of the stem may play a role in the development of end-of-stem pain.

## 1. Introduction

Revisions in total knee arthroplasty (TKA) are of rising importance. In the US, the amount of performed revisions rose by 39% from 2005 to 2010 [1]. Other countries with comprehensive registers, such as the UK, Australia and Sweden, also recorded increasing numbers of primary and revision TKAs [2–4]. In 2020, 23442 revision TKAs were performed in Germany [5]. This trend is expected to continue in the future. Schwartz et al. calculated the amount of revision TKAs from 2014 to 2030 in the US. They suggested a 78% to 182% increase [6]. The rising number of revisions leads to an increasing interest in complications of revision TKA.

In revision TKA, solid implant fixation plays an important role. Patients who underwent one or more revisions often suffer from a loss of bone density and quality [7, 8]. Reasons for this are the patient's primary disease, technical mistakes at the initial operation, aseptic loosening or infection, and the loss of bone mass due to the removal of the primary implant [9, 10]. Morgan-Jones et al. established a theory that describes how a stable implant fixation can be reached [11]. In their theory, the bone can be divided into three regions: the epiphysis or joint surface (zone 1), the metaphysis (zone 2), and the diaphysis (zone 3). A solid implant requires fixation in at least two zones [11]. As in revision TKA, the epi- and metaphysis often have significant bone defects, long diaphyseal stems are used to ensure fixation in zone 3. Discussion remains as to whether these stems should be cemented or should be implanted cementless as hybrid fixation. The hybrid fixation technique refers to a cemented fixation of the surface of the prosthesis and a cementless press-fit stem. While the use of cement allows the application of antibiotics and ensures an immediate, solid fixation, press-fit stems enable easier removal of the prosthesis in case of rerevision and do not require the insertion of bone cement into the medullary canal with increased risk of thromboembolism [12]. A recent meta-analysis by Sheridan et al. considered comparative studies of the last ten years and detected a significant lower failure rate of the hybrid fixation in comparison to the cemented fixation [13]. Thus, the hybrid fixation technique can be considered the preferred option according to current evidence. Nevertheless, some authors suggest that the hybrid fixation technique might come along with an increased risk of end-of-stem pain [8, 14, 15]. End-of-stem pain is localized pain at the tip of the stem of a prosthesis. The information about its prevalence varies. Sah et al. and Peters et al. reported only 0–2.3% end-of-stem pain after revision TKA [8, 16]. Other studies observed prevalences up to over 20% at the tibia and over 10% at the femur [14, 15, 17, 18]. For example, in Barrack et al.'s study, 11.6% of patients had end-of-stem pain on the femoral side when a cobalt-chrome stem was used [17]. The pain is described as mostly activity related [14, 17]. Although in some studies, the pain resolved spontaneously within a year, in other studies, the patients suffered from the pain throughout the whole observation period [15, 17]. Patients with end-of-stem pain are dissatisfied with the result of the operation and feel restricted in their daily activities [14, 15]. Unfortunately, there is no widely accepted treatment option. In cases with persisting pain, rerevision is sometimes unavoidable.

In the clinical studies that have been conducted yet, no significant correlation between stem diameter, stem length, and percentual canal fill with end-of-stem pain was found [14, 17, 18]. In a study by Barrack et al., patients with solid cobalt-chrome stems had significantly more pain than patients with slotted titanium stems, which the authors contributed to the lower elastic modulus of titanium compared to cobalt-chrome, but the specific stem design also seemed to have an influence [17]. Completo et al. measured strains on nine synthetic tibiae after the implantation of a conventional stem and a stem with a stem tip with decreased elastic modulus. Significant differences were only present when a massive varus load was applied [19]. In another study, the authors created finite element models and calculated that the maximum strains and contact pressures in tibiae after revision TKA was present at the tip of the stem [20]. All this evidence points towards a biomechanical cause of the pain, resulting from the interaction of stem and bone. In our study, we want to understand this interaction by performing the first study about end-of-stem pain in revision TKA investigating human donors.

The specific objectives of our study were to find out (a) how the stem is positioned in the medullary canal and where the contact points between stem and bone are located, (b) how the bend of the femur and the thickness of the cortex changes due to implantation, and (c) whether these results are influenced by the diameter of the trial in comparison to the diameter of the final stem.

## 2. Methods

We performed an in vitro study using 16 fresh-frozen human femora from 8 donors. The specimens were provided by the Medical University of Vienna. Donors with preexisting knee implants, obvious bone diseases or diseases that can affect the bone structure were excluded. The data were treated anonymously, and the study was approved by the LMU Munich ethics committee (project no. 20-142 KB).

**Figure 1:**
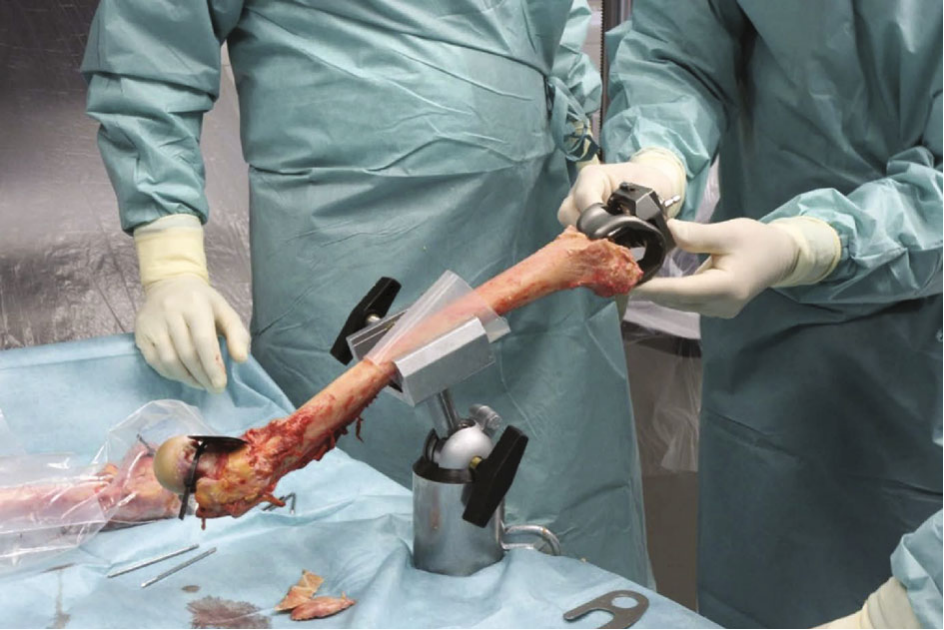
Implantation of a rotating hinge revision knee implant into 16 human femora.

A rotating hinge revision knee implant with a cobalt-chrome stem of 177 mm length (EnduRo modular rotating hinge knee system, Aesculap AG, Tuttlingen, Germany) was implanted into the femora by an orthopedic surgeon (Figure 1). The stem diameter (12–20 mm), the size of the femoral component (F2–F3), and the anterior-posterior shaft offset (±2 mm) were selected individually for each specimen. We used the hybrid fixation technique with a metaphyseal cementation and a cementless stem. During the reaming process, the surgeon's goal was to feel a resistance at the last 20–30 mm when inserting the trial, to ensure a gliding fit of this distance at the proximal stem tip.

In some knee revision systems, the trial has a smaller diameter than the final stem, when a press-fit stem is used. In order to find out whether this influences the implantation procedure and results, we compared two groups. In the standard group (*n* = 8), the implantation was performed according to the standard protocol. In the small_trial group (*n* = 8), the diameter of the trial was 0.5 mm smaller than the diameter of the final stem.

In order to minimize the influence of confounders, one femur of a donor was assigned to the standard group, while the contralateral femur was assigned to the small_trial group (Table 1).

**Figure 2:**
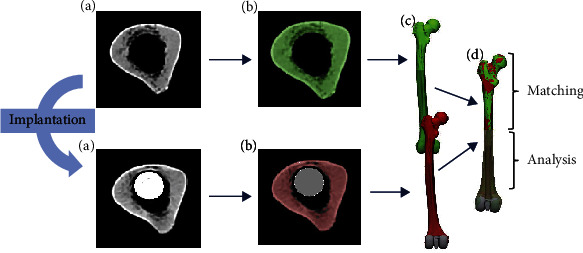
Workflow. (a) CT-scans of human femora before (top) and after (bottom) implantation of a rotating hinge knee implant. (b) Segmentation of cortex (green, red) and stem (grey). (c) Transformation into 3D-models. (d) Matching of the models.

A pre- and postoperative CT-scan was performed of each femur with a slice thickness of 1 mm, steps of 0.7 mm, and an average pixel size of 0.31 ± 0.03 mm (Somatom Perspective, Siemens AG, Munich, Germany) (Figure 2(a)). The scans were segmented using Mimics Medical 21.0 (Materialise NV, Leuven, Belgium) (Figure 2(b)), and 3-dimensional models of the femora were exported as Standard-Tessellated-Language (STL) surfaces (Figure 2(c)).

Mesh correction algorithms were applied in 3-matic 13.0 (Materialise NV, Leuven, Belgium). As the pre- and postoperative models lay differently in the virtual space, they had to be brought to spatial overlap without confounding the results. The models were aligned using the segmented proximal part of the pre- and postoperative model. The proximal parts were brought to maximal spatial overlap using 3-matic 13.0 (Materialise NV, Leuven, Belgium). The distal part of the bone (distal 150 mm of the stem and 50 mm proximal of the tip of the stem) was only moved passively along with the proximal part and thus was still available for analysis (Figure 2(d)).

The data were standardized to a right knee for data analysis. The implant was used as reference for the coordinate system (*z*-axis in the middle of the stem towards proximal, *x*-axis towards anterior, and *y*-axis towards medial). The origin lay 150 mm distal to the tip of the 177 mm long stem (the distal 27 mm of the stem was not analyzed, as in this region, the cortex was too thin for reliable segmentation).

**Figure 3:**
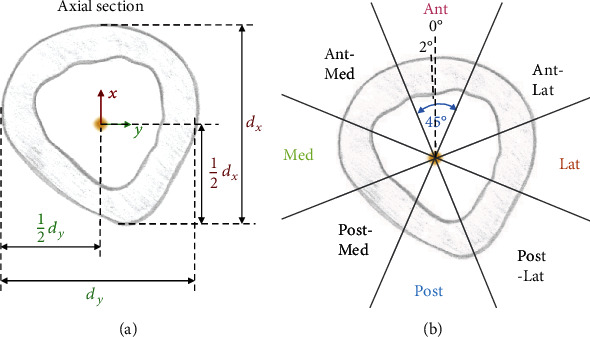
(a) Definition of the center point (yellow) on each cross section: midpoint between the anterior and posterior outer cortex wall and medial and lateral outer cortex wall in the cross section, *d*_*x*_: distance between anterior and posterior cortex wall, *d*_*y*_: distance between medial and lateral cortex wall. (b) Analysis performed along lines through the center point in 2° steps. Sectors: Ant: anterior; Ant-Lat: anterolateral; Lat: lateral; Post-Lat: posterolateral; Post: posterior; Post-Med: posteromedial; Ant-Med: anteromedial.

Cross sections of the models (perpendicular to the local *z*-axis) at different *z*-levels were created from the information of the STL-models (triangle vertices and normals), and the differences between the pre- and postoperative cross sections were calculated (MATLAB 2020a, MathWorks, Natick, USA). A schematic cross section of a postoperative femur is depicted in Figure 3.

The following analyses were performed for each cross section:

- *Position of the Stem in the Medullary Canal*. The distance between stem and inner cortex was calculated in a direction perpendicular to the stem surface
- *Change in cortical thickness*
- *Deformation of the Bone*. For this, the displacement vectors between the pre- and postoperative center points were calculated

For each cross section, the center point was defined as the midpoint between the anterior and posterior outer cortex wall and medial and lateral outer cortex wall in the cross section (Figure 3(a)).

The measurement of cortical thickness and displacement of the bone was performed along lines through this center point in 2° steps, and the mean was calculated within eight sectors of 45° (Figure 3(b)).

The basis for the statistical analysis was the mean values of the results obtained in the cross sections in different regions of the bone. The regions were defined as following: axial height 0 mm to <60 mm, 60 mm to <120 mm, 120 mm to <135 mm, 135 mm to <150 mm, 150 mm to <165 mm, and 165 mm to 200 mm. The axial height 0 mm defines the distal beginning of the analysis, the tip of the stem is at 150 mm, and the end of the analysis is at the axial height 200 mm.

The statistical analysis was performed using Statistica 10 (StatSoft Europe GmbH, Hamburg, Germany). An analysis of variance (ANOVA) was conducted to test for significant differences between the standard group and the small_trial group with a significance level of *p* < 0.05. Before the analysis, the normal distribution of the data (normal *p* − *p* plots; *p* < 0.05) and the homogeneity of variance (Levene-test) were verified. After this, the Scheffe-Test was carried out as post hoc test.

## 3. Results

### 3.1. Position of the Stem in the Medullary Canal

The most important contact between stem and bone was found at the proximal 30 mm of the stem. There, the contact was especially anterior, but also medial and lateral. In these three sectors, the distance between stem and cortex gradually decreased towards the tip of the stem.

**Figure 4:**
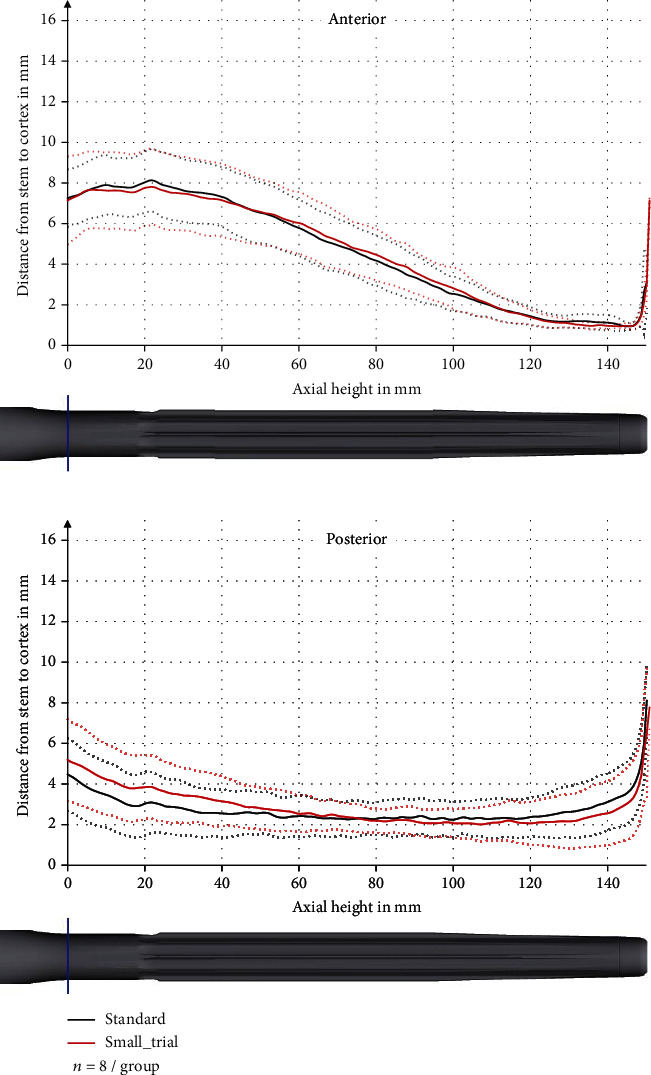
Distance between stem and cortex in the anterior and posterior sector with pooled standard deviation (dashed line).

**Figure 5:**
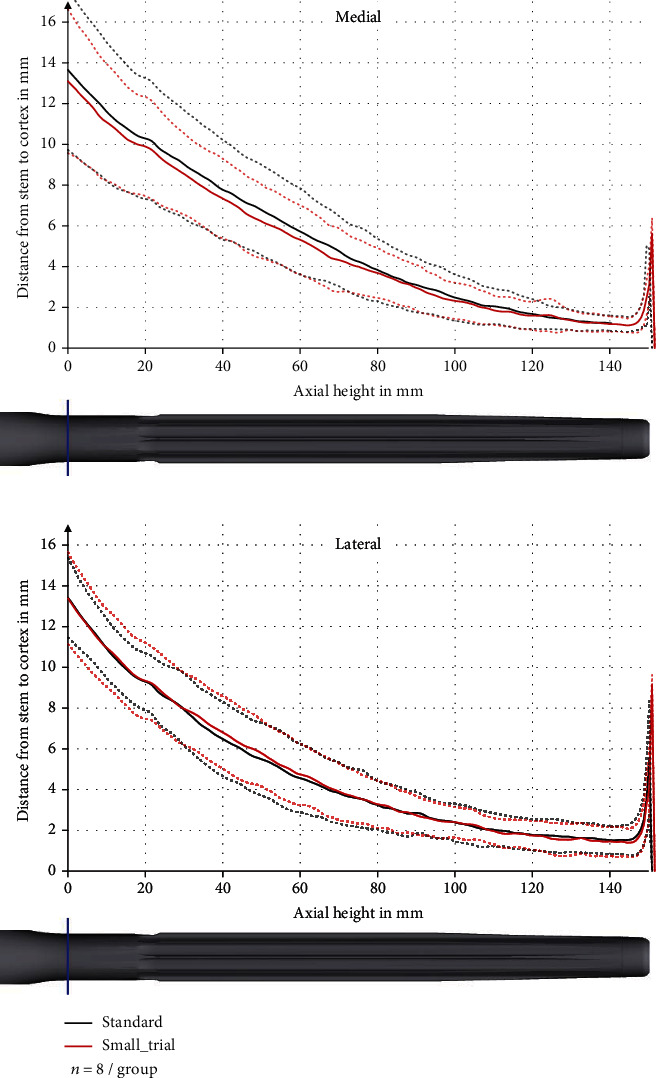
Distance between stem and cortex in the medial and lateral sector with pooled standard deviation (dashed line).

The minimum average distance between stem and cortex was 0.95 ± 0.14 mm at the axial height of 146 mm anterior, 1.15 ± 0.35 mm at the axial height of 145 mm medial, and 1.46 ± 0.71 mm at the axial height of 144 mm lateral. Posterior, the main contact was found to be at the middle of the stem with a minimum average distance between stem and cortex of 2.16 ± 0.80 mm at the axial height of 100 mm (Figures 4 and 5). There was no significant difference in distance between stem and cortex between the two compared groups (*p* values between 0.27 and 0.99).

We discovered different patterns of how the straight stem can lay in the bowed femur. The main difference between the patterns is the anterior and posterior contact points, which can be seen in the sagittal section.

**Figure 6:**
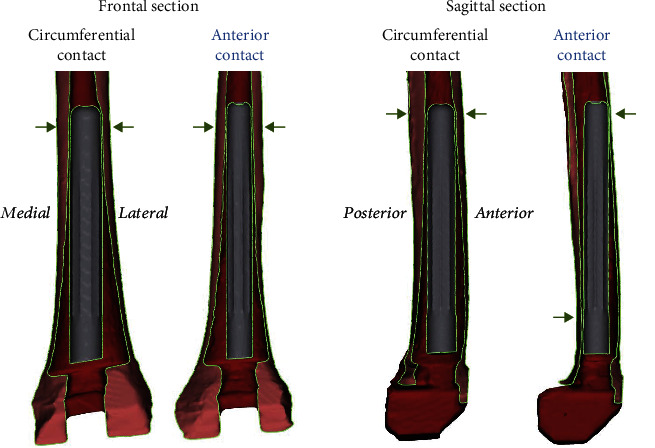
Contact patterns: exemplary specimen with circumferential contact between the tip of the stem and the cortex (ID: small_trial_6) and exemplary specimen with only anterior contact between the tip of the stem and the bone (ID: small_trial_7).

Specimens with the circumferential contact pattern had a circumferential interaction between stem and bone at the tip of the stem. An example for this can be seen in Figure 6. In contrast to this, in other specimens, the tip of the stem only had contact with the anterior cortex but no contact posterior, which we called the anterior contact pattern (Figure 6). There were graduations and therefore also some specimens that could not be classified into one of the two groups. In five donors, both autologous femora had the same contact pattern (Table 2).

### 3.2. Change in Cortical Thickness

**Figure 7:**
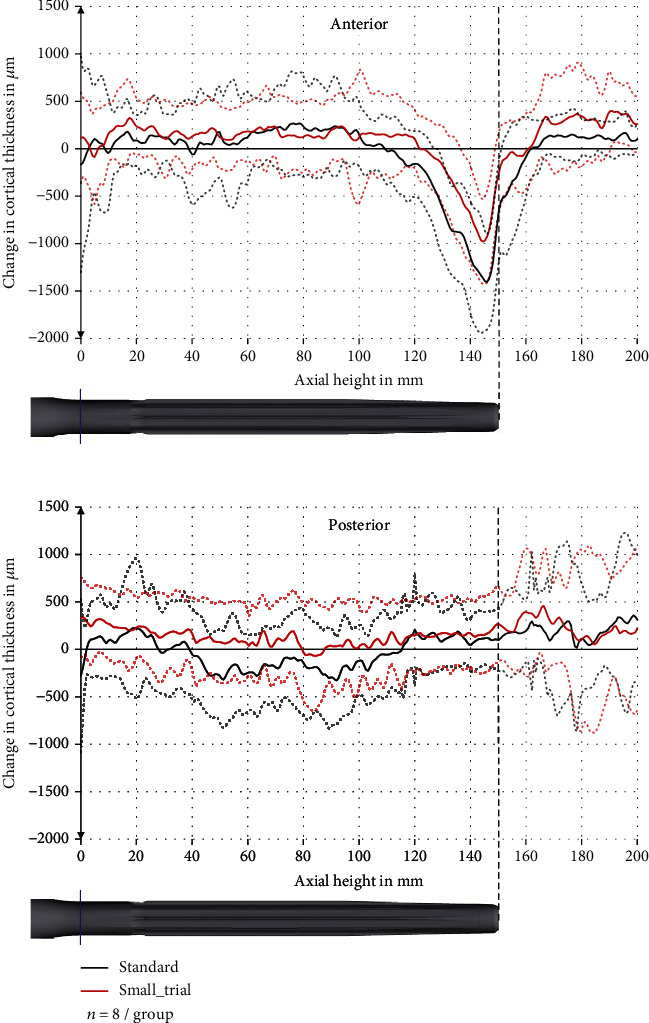
Change in cortical thickness in the anterior and posterior sector with pooled standard deviation (dashed line).

The cortical thickness was decreased especially at the tip of the stem (axial height 120–160 mm) and predominantly anterior. The maximum decrease anterior was 1405 ± 501 *μ*m (standard deviation) at the axial height 146 mm in the standard group and 980 ± 447 *μ*m at the axial height 145 mm in the small_trial group (Figure 7). This is a relative reduction in comparison to the preoperative cortical thickness of 34 ± 14% and 26 ± 14%, respectively.

In the region 120 mm to <135 mm, the anterior reduction in cortical thickness was significantly higher in the standard group than in the small_trial group (*p* = 0.046) with a reduction of 481 ± 328 *μ*m and 129 ± 314 *μ*m, respectively. There were no significant differences in the other regions.

**Figure 8:**
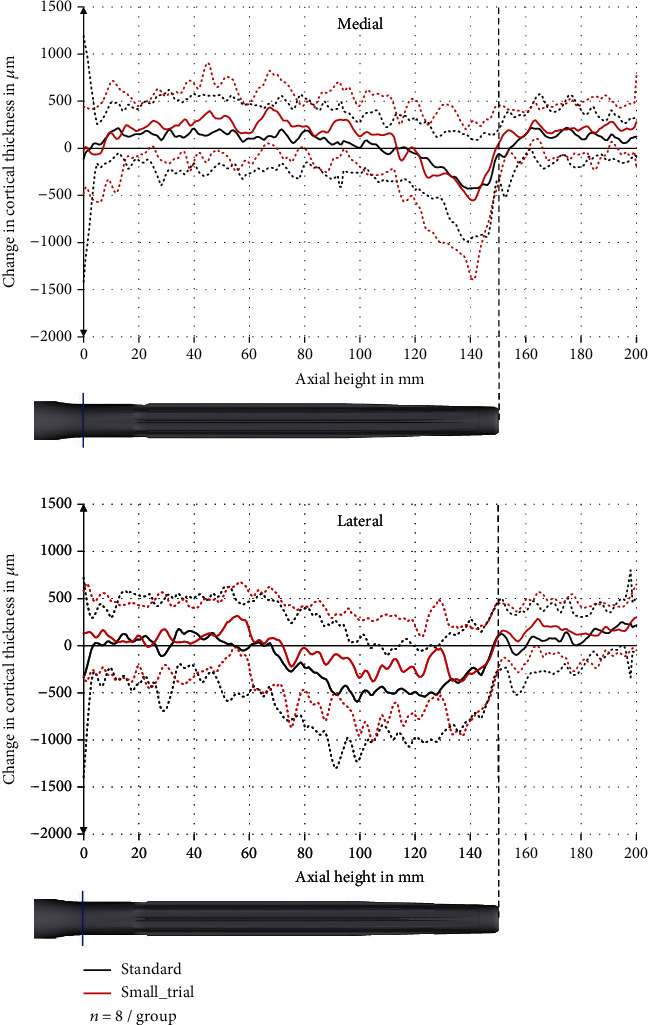
Change in cortical thickness in the medial and lateral sector with pooled standard deviation (dashed line).

Posterior, there was only a slight reduction in cortical thickness in the middle of the stem (axial height 40 mm to 110 mm) (Figure 7). Medial and lateral, the cortical thickness was only reduced slightly (Figure 8).

### 3.3. Deformation of the Bone

**Figure 9:**
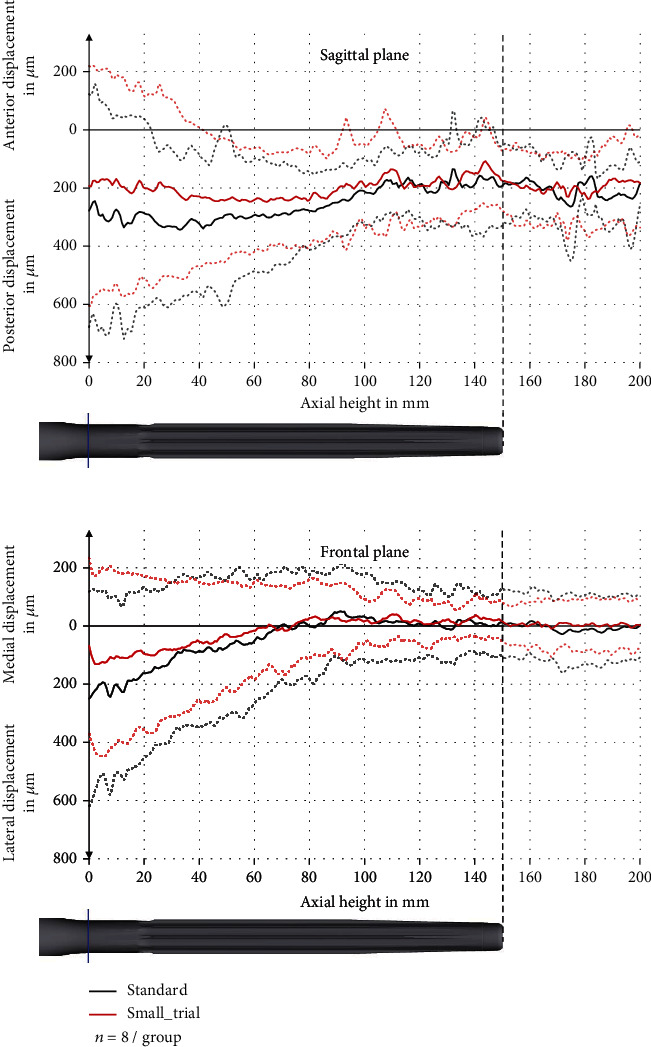
Displacement in the sagittal and frontal plane with pooled standard deviation (dashed line).

Our results showed a combined elastic and plastic deformation of the bone resulting from the implantation. The axis of the bone was displaced to posterior and lateral with an increasing displacement from proximal to distal. The maximum displacement in the standard group was 346 ± 240 *μ*m to the posterior and 250 ± 368 *μ*m to the lateral side. The maximum displacement in the small_trial group was 248 ± 166 *μ*m to the posterior and 131 ± 301 *μ*m to the lateral side. The differences were not significant (*p* > 0.53) (Figure 9).

## 4. Discussion

The objectives of our study were to find out (a) how the stem is positioned in the medullary canal and where the contact points between stem and bone are, (b) how the femoral geometry (bend of femur and cortical thickness) changes due to the implantation, and (c) whether these results are influenced by the diameter of the trial in comparison to the diameter of the final stem. In order to solve these questions, we performed an in vitro study. We compared 3-dimensional bone models that were derived from CT-scans of 16 human femora before and after the implantation of a revision knee implant.

To our knowledge, this is the first study that analyses the biomechanical changes of the femur after stemmed total knee arthroplasty. Therefore, we cannot compare our results to other studies. We would like to emphasize that we used human specimens and that the implantations were performed by a senior orthopedic surgeon using the system routinely for his patients. Hence, we aim to deliver a reliable transferability of our results to the clinical situation.

We decided for a preclinical study design. Consequently, it was not known which of the specimens would suffer from end-of-stem pain. On the other hand, the preclinical design came with the possibility of further analyses. For example, we were able to measure surface strains on the investigated femora under dynamic load. These results will be published in a separate publication. Additionally, the method that was implemented in this study might be applied in future clinical studies using CT-scans of patients.

Our study showed that the main contact between stem and bone lay at the tip of the stem. Different contact patterns between stem and bone were observed, a phenomenon for which the discrepancy between the straight stem and the bowed femur is the main factor of influence. Furthermore, the entry point and angle of the reamer may play an important role in the determination of the resulting contact pattern. If the entry point lies anteriorly, the circumferential contact pattern might result. If the entry point lies more posteriorly, the anterior contact pattern may result more likely (Figure 6). As in five donors, both autologous femora had the same contact pattern (∗ in Table 2), it can be concluded that the contact pattern depends especially on the anatomical conditions and less on the implantation method (standard or small_trial). The relevance of these different contact patterns has yet to be determined. Although Albino et al. found no correlation between a lack of parallelism between stem and cortex in radiographs and tibial end-of-stem pain, there should be awareness of the different contact patterns in the human femur [18]. All specimens had a 3-point contact between stem and cortex with contact at the epiphysis and at least two contact points at the diaphysis (Figure 6). Therefore, both groups (standard and small_trial) and both contact patterns (circumferential and anterior) can be considered a stable fixation and we do not expect loosening in either case.

In addition to that, we saw a decrease in the cortical thickness at the tip of the stem, especially anterior. The decrease was up to 34% in the standard group and up to 26% in the small_trial group. This reduction in cortical thickness might further decrease the mechanical stability of the femur at the tip of the stem and increase the surface strains that might lead to end-of-stem pain.

It should be mentioned that the specimens in the small_trial group experienced less reduction in cortical thickness, even though this was only significant in one region (120 mm to >135 mm, anterior). The reason for this difference might be that in the standard group, the trial had a larger diameter and gave the surgeon the feeling of a tight fit of the stem, which might have led to a more aggressive reaming. This underlines the role of the surgeon and the instruments during the implantation. Apart from this, a 0.5 mm smaller trial (small_trial group) does not seem to influence the implantation significantly. Therefore, we currently do not consider the trial size as main factor for the development of end-of-stem pain.

We also figured out that the implantation of a stiff stem into human femora leads to a combined elastic and plastic deformation of the bone to posterior and lateral. The exact mechanism of all possible factors influencing the deformation cannot be stated at the current point of research. We hope that following FEM-simulations will give further information about the reason and effect of femoral bowing. Summing up, the detection of a combined elastic and plastic deformation of the bone shows that the impact of a long, stiff stem should not be underestimated and should be considered during the development and further optimization of new stem designs.

The changes in bone morphology that were found in this study indicate that the tip of the stem should be seen as an important biomechanical region. In addition to the theories that have already been pointed out in the literature, different contact patterns between the tip of the stem and the cortex (circumferential or anterior contact), as well as a reduction in cortical thickness may play an important role in the development of end-of-stem pain.

From our observations, we conclude that there is no significant difference between the standard implantation and the small_trial group with a slight tendency of less bone displacement and less cortical bone reduction when the diameter of the trial is reduced in comparison to the final stem (small_trial group).

In the next steps, the surface strains on the investigated femora will be measured under dynamic load in order to find out whether high surface strains are present at the tip of the stem. Furthermore, the 3-dimensional models that were created in this study will serve for a FEM-model that allows to calculate the surface strains on human femora while investigating different stem designs and materials.

## Figures and Tables

**Table 1:** Overview of the specimens (*n* = 16).

Donor	ID	Knee	Group	Femoral component	Diameter of trial (mm)	Diameter of stem (mm)
1	Small_trial_1	Left	Small_trial	F3	13,5	14
	Standard_1	Right	Standard	F3	14	14

2	Standard_2	Left	Standard	F2	12	12
	Small_trial_2	Right	Small_trial	F2	12,5	13

3	Small_trial_3	Left	Small_trial	F3	15,5	16
	Standard_3	Right	Standard	F3	16	16

4	Small_trial_4	Left	Small_trial	F3	17,5	18
	Standard_4	Right	Standard	F3	17	17

5	Standard_5	Left	Standard	F2	15	15
	Small_trial_5	Right	Small_trial	F2	14,5	15

6	Standard_6	Left	Standard	F3	16	16
	Small_trial_6	Right	Small_trial	F3	15,5	16

7	Standard_7	Left	Standard	F3	20	20
	Small_trial_7	Right	Small_trial	F3	19,5	20

8	Small_trial_8	Left	Small_trial	F3	19,5	20
	Standard_8	Right	Standard	F3	20	20

**Table 2:** Classification into contact patterns. Asterisk∗: autologous femora with same contact pattern.

ID	Contact pattern
Small_trial_1∗	Circumferential
Standard_1∗	Circumferential
Standard_2∗	Anterior
Small_trial_2∗	Anterior
Small_trial_3∗	Circumferential
Standard_3∗	Circumferential
Small_trial_4	Circumferential
Standard_4	Between both patterns
Standard_5	Anterior
Small_trial_5	Between both patterns
Standard_6∗	Anterior
Small_trial_6∗	Anterior
Standard_7	Anterior
Small_trial_7	Circumferential
Small_trial_8∗	Anterior
Standard_8∗	Anterior

## Data Availability

The data used to support the findings of this study are available from the corresponding author upon request.
